# A 3D Image Filter for Parameter-Free Segmentation of Macromolecular Structures from Electron Tomograms

**DOI:** 10.1371/journal.pone.0033697

**Published:** 2012-03-29

**Authors:** Rubbiya A. Ali, Michael J. Landsberg, Emily Knauth, Garry P. Morgan, Brad J. Marsh, Ben Hankamer

**Affiliations:** Institute for Molecular Bioscience, The University of Queensland, St Lucia, Queensland, Australia; Université Joseph Fourier, France

## Abstract

3D image reconstruction of large cellular volumes by electron tomography (ET) at high (≤5 nm) resolution can now routinely resolve organellar and compartmental membrane structures, protein coats, cytoskeletal filaments, and macromolecules. However, current image analysis methods for identifying *in situ* macromolecular structures within the crowded 3D ultrastructural landscape of a cell remain labor-intensive, time-consuming, and prone to user-bias and/or error. This paper demonstrates the development and application of a parameter-free, 3D implementation of the bilateral edge-detection (BLE) algorithm for the rapid and accurate segmentation of cellular tomograms. The performance of the 3D BLE filter has been tested on a range of synthetic and real biological data sets and validated against current leading filters—the pseudo 3D recursive and Canny filters. The performance of the 3D BLE filter was found to be comparable to or better than that of both the 3D recursive and Canny filters while offering the significant advantage that it requires no parameter input or optimisation. Edge widths as little as 2 pixels are reproducibly detected with signal intensity and grey scale values as low as 0.72% above the mean of the background noise. The 3D BLE thus provides an efficient method for the automated segmentation of complex cellular structures across multiple scales for further downstream processing, such as cellular annotation and sub-tomogram averaging, and provides a valuable tool for the accurate and high-throughput identification and annotation of 3D structural complexity at the subcellular level, as well as for mapping the spatial and temporal rearrangement of macromolecular assemblies *in situ* within cellular tomograms.

## Introduction

Electron tomography (ET) is an important tool for studying structural cell biology *in situ* by bridging the resolution gap between light microscopy and methods for protein structure determination at atomic resolution, such as X-ray and electron crystallography as well as nuclear magnetic resonance (NMR) spectroscopy. Recent advances in ET at the level of sample preparation, improved detector sensitivity/capture efficiency and imaging resolution, along with automated computational techniques for 3D image reconstruction, processing and analysis now enable macromolecular assemblies to be resolved at up to 15–30 Å, in the best case examples [1]. Meanwhile, the 3D reconstruction of extremely large cytoplasmic volumes at ∼3–6 nm resolution and even entire mammalian cells at ∼10 nm resolution by cellular ET now affords unprecedented new insights regarding the structure-function relationships that exist among subcellular compartments/organelles, the plasma membrane, cytoskeletal filaments, large macromolecular assemblies as well as membrane proteins [2]–[5]. 3D cellular reconstructions of this nature thus provide a precise spatial framework for developing annotated, pseudo-atomic resolution 3D atlases of cells through docking high resolution structures of macromolecular assemblies.

A critical step in the advancement of molecular resolution ET is the ability to accurately segment molecular structures *in situ* within cellular tomograms. Classical edge-detection algorithms such as the Sobel [6], Prewitt [6], Laplacian of Gaussian [6] and Canny edge detectors [7] are increasingly being incorporated into semi-automated and automated methods for segmenting 3D image volumes. However, all of these are best suited to images with relatively high signal-to-noise ratios (SNR) and thus have limited use for the accurate/automated analysis of cellular tomograms, which have an inherently low SNR. By comparison, more modern filters [8]–[16] exhibit improved edge-detection performance at low SNR. However, for the most part these algorithms have only been implemented in 2D and thus have limited utility for analysing 3D image volumes. A true 3D filter, capable of using data from adjacent slices, offers the advantage that additional information from either side of the ‘focal’ slice can be considered, thereby enabling enhanced noise suppression along with the detection of contiguous and legitimate structural details throughout the 3D image stack.

The Canny edge detector [7], [17] is widely considered to be a “gold standard” filter [18] for 2D analysis. More recently it has been implemented in 3D (http://www.imagescience.org/meijering/software/featurej/edges.html). This implementation is a multi-stage, complex filter, which in principle involves four fundamental steps. In the first step it convolves the target volume with a Gaussian filter to smooth the image and suppress the noise. The second step calculates gradients of the image using a Sobel edge detector, the rationale of applying which is to identify voxels with sufficiently large weighting magnitudes that identify them as an edge. In step three, non-maximum peak suppression is performed to track the edge points along the high magnitude regions and to eliminate the remaining voxels. This is followed by step four, which through hysteresis thresholding converts the output volume into a binary format to ensure that noise voxels are not included as part of a true edge.

Optimization of the filter's performance requires the simultaneous fine-tuning of three parameters: the standard deviation of the Gaussian as well as the high and low hysteresis thresholds. The need to simultaneously optimize multiple parameters makes the use of the 3D Canny labor-intensive and impractical for application to high throughput, automated or semi-automated analysis. An additional drawback is that it fails to detect true discontinuities (i.e. it is unable to discriminate between contrast discontinuities that are due to noise or a true edge).

The 3D recursive filter [19] offers a simplified alternative to the 3D Canny filter. It approximates the gradient of an image by computing the impulse response recursively and finally applies a pseudo 3D edge-closing algorithm; that is, it uses a 2D edge-tracking algorithm [20] that is applied to each XY, XZ and YZ plane separately. In practice, the tracking algorithm, which is designed to complete discontinuous contours within a 2D plane, limits its ability to accurately detect the 3D structure of an object.

The underlying principles of the bilateral edge filter [16] offer an attractive alternative to the Canny and 3D recursive edge-detection filters. The BLE is a nonlinear, photometrically-weighted, discontinuity-based anisotropic filtering technique that has been shown to be suited to images containing predominantly low- and mid-frequency information [16]. More specifically, it suppresses noise by attenuating undesired frequencies and enhances edge-detection by selectively extracting specific features. However it still requires user modification of the manual parameter (σ_2_) and has not been implemented in 3D.

In this paper, we present a full 3D implementation of the bilateral edge filter (3D BLE), which importantly also eliminates the need for manual σ_2_ optimization. This fully automated 3D BLE is a simple and fast filter, specifically designed for electron tomography data which typically has low signal to noise ratios, but also suitable for analysis of a wide range of other 3D data. Our implementation includes Gaussian filtration followed by iterative median filtration as a pre-filtering step [6], [21]. The iterative median filter maintains edges and converges to an optimal solution beyond which it does not keep smoothing. This significantly improves edge-detection by simplifying the voxel intensity distribution and suppressing noise disturbances without corrupting the edge information. The output 3D binary image data can then be used together with automated segmentation, or for edge-detection-based 3D particle picking.

## Results and Discussion

### Adaptation of the 2D bilateral edge filter to analysis of 3D image volumes

In this paper, the original 2D variant of the BLE filter [16] has been extended to operate in three dimensions. Additionally, parameter adjustment has been fully automated. Similar to the 2D BLE filter, the 3D BLE filter first calculates the photometric score for each individual voxel (focal voxel) in the context of the ‘processing window’ of the image volume being analysed. A score of 0 represents a perfect edge while 1 represents noise, for each individual voxel. The rationale of sequentially calculating photometric scores for each focal voxel, as the window moves across the image, is to build a photometric score map, from which edges can be traced. The photometric function 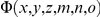, corresponding to an adjacent neighboring voxel 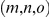 from the focal voxel 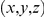 is defined in Eq 1.
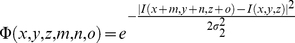
(1)



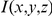
 is the original volume and 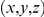 indicates the coordinates of the focal voxel, while 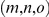 indicates the adjacent coordinates of a neighboring voxel to the focal voxel (which can be expressed as: m = {x−1, x, x+1}, n = {y−1, y, y+1} and o = {z−1, z, z+1}). 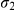 is a photometric parameter which defines the minimum difference in intensity that is to be regarded as an edge. In the 2D BLE, 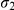 is the only parameter requiring manual adjustment. While manual adjustment ensures the highest quality result as it allows the fine tuning of σ_2_, in this 3D implementation the user can choose to adjust this parameter automatically to achieve high thoughput, by replacing it with the actual standard deviation (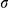) of the volume (Eq. 2):
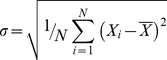
(2)where *N* is the total number of voxels, 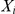 is the current voxel intensity and 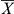 is the mean value of all the intensities present in the volume. Automated optimisation of this photometric parameter is highly desirable to facilitate segmentation of large and complex cellular tomograms in a high throughput manner.

Voxels having intensities above the background level may correspond to signal or noise and the further such a high intensity voxel is from a focal voxel, located on a given edge, the less likely it is to be part of that edge. Consequently, to distinguish between edge and noise voxels, each photometric score of a neighboring voxel 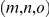 is next spatially weighted according to a 3D Gaussian distribution centred at the focal voxel (i.e. the further a voxel of a given intensity is from the focal voxel, the lower its photometrically-weighted score). The Gaussian weightings 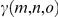 are given in Eq. 3.
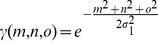
(3)


Here 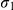 is used to define the significant weights and is fixed to 2 voxels, similar to the 2D case [16]. It is also used to determine the size of the neighborhood for the calculation of the normalized photometric score given in Eq. 4.

To identify substantial discontinuity (i.e. an edge), the photometric score is next normalized by averaging across a pre-defined number of voxels (see Eq. 4). The normalized photometric score 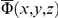 for the given focal voxel is calculated as a Gaussian weighted average of the individual photometric scores over a radius of 2 voxels.
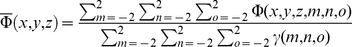
(4)


The normalized photometric scores indicate the significance of discontinuity of a given edge. Scores close to 1 are considered weak photometric responses and can correspond to fluctuations in background noise; single, high intensity, spurious voxels or the focal voxel otherwise not being centered on an edge. Strong scores (close to 0) represent voxels that are likely part of an edge. After analysing the possible numerical representations of connectivity of an edge, a threshold of 0.85 was chosen. This setting was repeatedly found to provide the largest observed gain in edge-detection performance. Average photometric scores below this threshold are considered to indicate edge voxels and scores greater than 0.85 indicate spurious voxels, non-edge voxels, or weak fluctuations in background intensity due to anisotropy. The thresholded photometric score 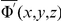 is thus given by:
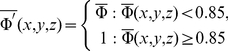
(5)


Consequently 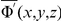 remains unaltered if its value is below 0.85, but is adjusted to 1 if its value is greater than or equal to 0.85. The lowest scores in the final photometric map generated by Eq. 5 represent local minima or troughs along which an edge is traced based on the use of a 3×3×3 voxel volume. A focal voxel in a 3×3×3 volume (i.e. 27 voxels) therefore has the possibility of forming 26 different connections with its immediate neighbors. If the focal voxel is part of a continuous trough (edge) or at the end of a trough, its average photometric score will be within the thirteen smallest scores of a (3×3×3) voxel neighborhood. This is because twelve represents the number of voxels required to describe a continuous edge within this 3D volume of 27 voxels (4 voxels within each plane of a 3×3×3 volume = 12 voxels). Increasing this value tends to increase edge connectivity further in noise free data, but in the presence of high levels of noise (e.g. cryo-EM data) this tends to result in decreased noise suppression. Theoretically, a continuous edge of 5 voxels within a 3×3 voxel plane ( = 15 voxels in a 3×3×3 volume) is possible. However thresholding at a value of 15 also allows edges with branching (or noise contamination) to be detected. Thirteen in our experience is a sensible compromise (see Supplementary Material of Pantelic *et al.* [16] for further explanation).

### Application to synthetic data

**Figure 1:**
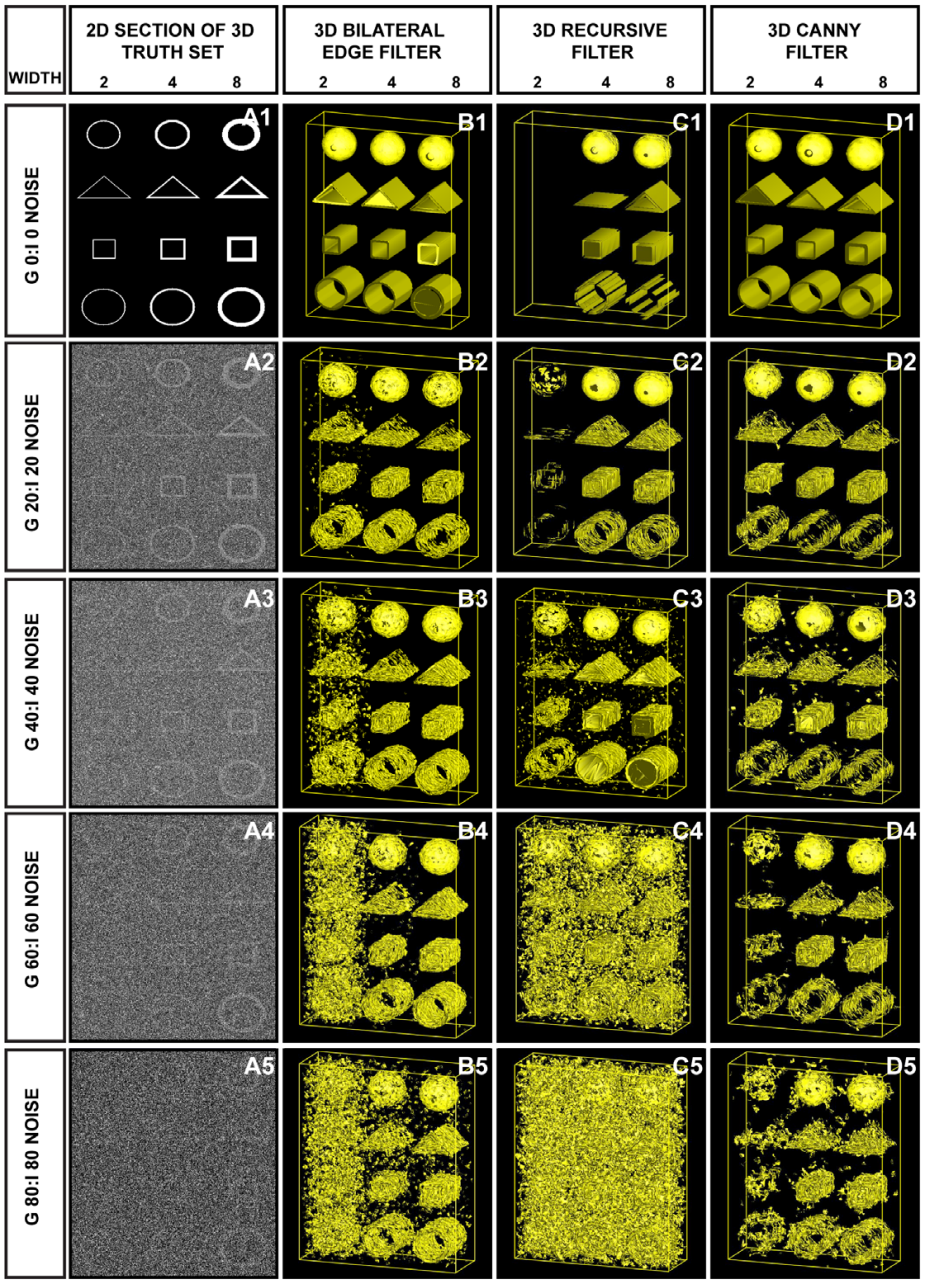
Application of 3D BLE to synthetic phantoms corrupted with Gaussian and impulse noise. Performance of the 3D BLE, 3D recursive and 3D Canny filters was assessed using a volume of 3D synthetic phantoms contaminated with increasing levels of Gaussian and impulse noise. (**A1–A5**) 2D sections taken from synthetic volumes contaminated with increasing levels of Gaussian and impulse noise. (**B1–B5**) 3D surface rendering of results (B1–B5) obtained from the 3D BLE filter. (**C1–C5**) Surface rendering of the 3D recursive-filtered synthetic dataset. (**D1–D5**) Surface rendering of the 3D Canny-filtered synthetic dataset.

Using a combination of real and synthetic datasets, the performance of the 3D BLE filter was evaluated and compared to two benchmark 3D edge-detection algorithms: the 3D Canny and 3D recursive filters. To obtain a fair comparison, all test volumes in the initial phase were subjected to the same pre-filtering step and the recommended or default settings of each filter were used. No attempts at parameter adjustment were made. As a first test, the noise suppression and edge-detection abilities of the 3D BLE filter were evaluated using synthetic “truth” reference volumes (hollow cylindrical, spherical, triangular and rectangular) contaminated with different combinations of Gaussian (G) and impulse (I) noise (Figure 1). Results were evaluated based on three criteria: response to true edge directionality, the minimum detectable object edge width (in pixels) and capability to detect true edges and distinguish them from noise in images corrupted with high levels of noise (Figure 1, Table 1). Such an analysis enables the quantification of the minimum possible signal to noise ratio (SNR) required for the filter to detect edges.

**Table 1:** Statistical evaluation of filter performance using synthetic volumes contaminated with different levels of Gaussian and impulse noise shown in Figure 1.^a^

NoiseGaussian/Impulse	3D BLE (%)	3D recursive (%)	3D Canny (%)
**0/0**	**29.66** (1.35)	**30.03** (1.39)	**26.91** (1.11)
**20/20**	**46.24** (3.20)	**118.46** (21.58)	**38.52** (2.28)
**40/40**	**52.52** (4.20)	**120.74** (22.42)	**42.05** (2.72)
**60/60**	**63.36** (6.17)	**123.00** (23.27)	**43.73** (2.94)
**80/80**	**67.86** (7.08)	**124.96** (24.01)	**45.62** (3.20)

a RMSE scores between the input volumes and the three filter outputs are shown in bold. Smaller scores represent higher levels of correlation with the input volume. Values in brackets are the percentage voxel variation between input volumes and the three filtered outputs.

Noise was introduced incrementally from an initial value of 5% G/5% I (expressed as a percentage of the signal intensity) up to 80% G/80% I. The width of the reference objects was varied from 1 to 16 pixels. Figure 1 shows a subsample of the tests performed that highlight the performance limitations of the 3D BLE (Figure 1B) in comparison to the 3D recursive (Figure 1C) and Canny filters (Figure 1D). Post-processed 3D surface views of the test datasets clearly highlight performance differences between the three filters. At low noise levels (G 0/I 0 to G 20/I 20) the three filters performed similarly and effectively detected the edges of all reference volumes. However as the noise levels increased further (G 40/I 40 to G 80/I 80) differences in performance emerged. At G 60/I 60 or greater, the 3D BLE was unable to effectively discriminate the structure of the object from noise at an edge width of 2 pixels, but was able to recognise all test objects with an edge width of 4 pixels or greater even at G 80/I 80. Corruption with G 80/I 80 noise provided a stringent test in which it was almost impossible to distinguish the objects from noise by eye (Figure 1 A5) but the 3D BLE was still capable of significantly amplifying object information above the level of the noise for edge widths of 4 and 8 pixels. By comparison, the capabilities of the 3D recursive filter appear to be limited at G 60/I 60 and beyond, regardless of edge width. Furthermore at the lower noise levels of G 40/I 40 and even G 20/I 20 the 3D recursive filter, while effectively dampening background noise, poorly resolved edges 2 pixels in width in comparison with the 3D BLE and the Canny filters. Consequently we concluded that the performance of the 3D BLE filter was better than that of the 3D recursive filter and particularly so at high noise levels.

A comparison of the performance of the 3D BLE filter with the Canny filter at G 60/I 60 and above indicated that the 3D BLE achieved a better edge connectivity at 4–8 pixel widths (see Figure 1 B4 vs. D4; B5 vs. D5). In the 2 pixel test the 3D BLE showed signs of noise contamination (Figure 1 B4 & B5) while the Canny filter showed poor connectivity (Figure 1 D4 & D5). At G 80/I 80, the Canny and 3D recursive edge detectors both treated high frequency noise voxels as edges and thus failed to highlight true structural information (see Figure 1 C5-D5). In contrast the 3D BLE filter, while retaining some high frequency noise voxels, appeared to better preserve edge detail (Figure 1 B5).

Collectively these tests suggest that the 3D BLE filter has a similar level of performance to the Canny filter. An important advance over the Canny, however, is that the 3D BLE filter achieves a similar level of performance for many test images of this type in an automated fashion, while the Canny requires the optimization of 3 parameters for each image. Parameter-free filtration is essential for automated segmentation.

Table 1 lists the respective root mean square error (RMSE) values calculated between the filtered volumes and the truth set. Analogous to Pantelic *et al.* [16], the truth image was constructed by applying the Canny edge-detector to the noise/CTF/envelope-free variant of Figure 1 A1. We then compared the truth image with the results generated by all filters (Figure 1 B1–5 to D1–5). The 3D BLE filter outperformed the recursive filter, yielding lower RMSE values in each case (see Table 1). However for all five tests, the Canny yielded the lowest RMSE scores. The apparently improved performance of the Canny filter under these conditions was not surprising due to the fact that the truth image was constructed based on the output of the Canny edge detector. The percentage voxel variation between the truth image and the filtered image was also calculated (see Table 1). The apparently improved performance of the Canny filter under these test conditions could be attributed to the more rapid fall off in edge-detection by the 3D BLE for thin objects (edge width of 2 pixels) compared with that of the Canny, where performance deteriorated almost equally, regardless of edge width. In summary, these tests indicated that the 3D BLE filter performed significantly better than the 3D recursive filter and similarly to the Canny filter under the test conditions analysed.

In the preceding tests, the 3D BLE filter was able to resolve contours of truth reference images from synthetic impulse and Gaussian noise at levels up to 80% of the signal intensity. In the next stage of testing the truth reference set was contaminated with noise more closely simulating “real” experimental conditions encountered in electron micrographs, in order to provide a more realistic evaluation of the performance of the 3D BLE filter for EM data.

**Figure 2:**
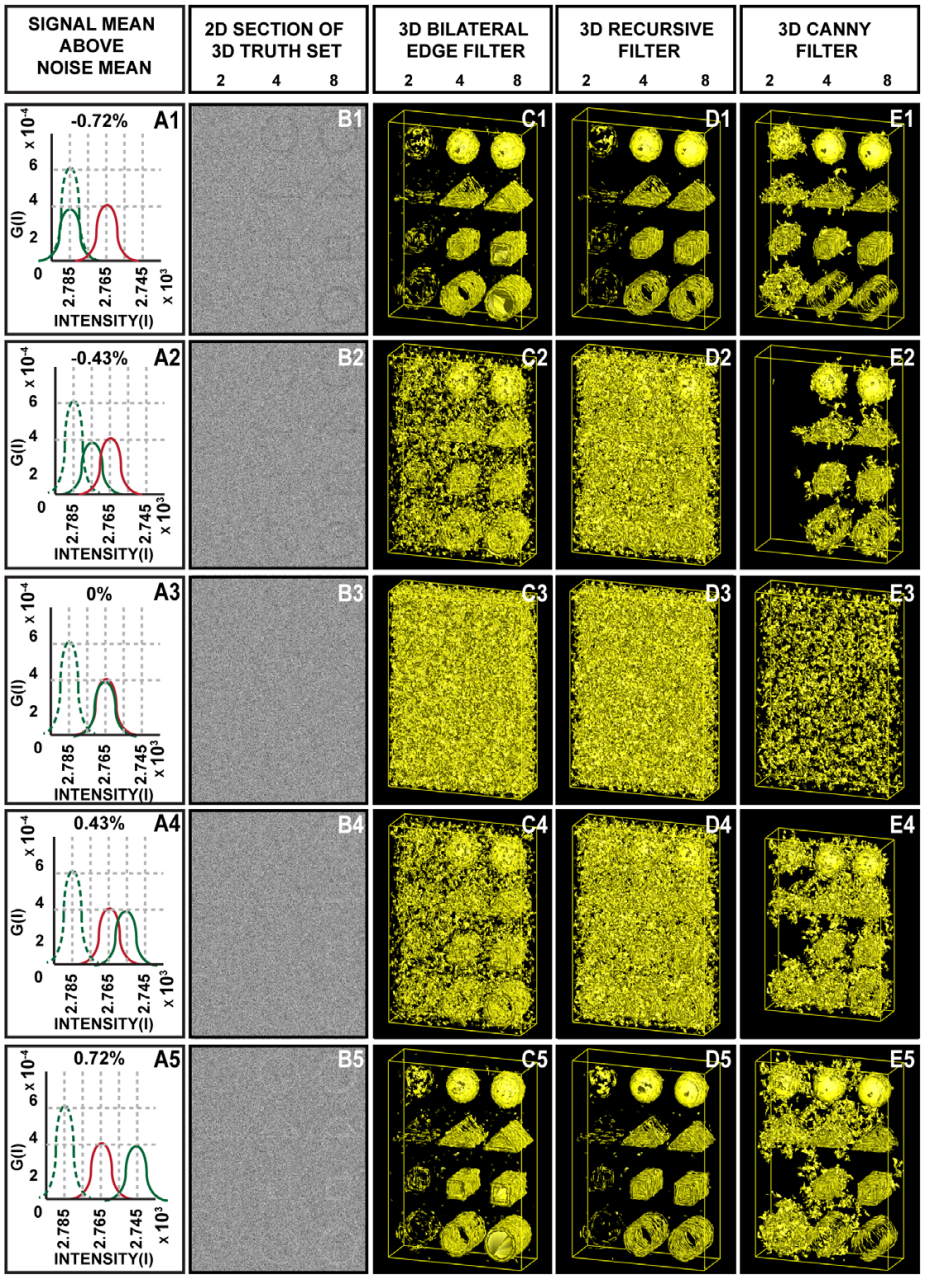
Application of 3D BLE to synthetic phantoms corrupted with simulated cytosolic noise. Performance of the 3D BLE, 3D recursive and 3D Canny filters was assessed using the same volume of 3D synthetic phantoms shown in Figure 1, but contaminated with different levels of simulated experimental noise. (**A1–A5**) A graphical representation of the SNR present in the five representative cases shown. Coloring in A1–A5 is as follows: **green dotted line** shows the contrast and intensity of the original signal; **red line** shows the contrast and intensity of the noise; **green solid line** shows the scaling and shifting of signal profile towards noise profile. Overall, the graph shows the probability density function (G(I)) of the normal distribution (**B1–B5**) 2D sections taken from synthetic volumes contaminated with experimental noise. (**C1–C5**) 3D surface rendering of results obtained following application of the 3D BLE filter to the synthetic dataset. (**D1–D5**) Surface rendering of 3D recursive-filtered test dataset. (**E1–E5**) Surface rendering of 3D Canny-filtered dataset.

The most stringent test settings used are shown in Figure 2 panels A3-E3. In this experiment the contrast and intensity of the original signal (A3 – dotted line) was adjusted to match that of the noise so that the signal (A3 - green line) and noise profiles (A3 - red line) were identical in contrast. Consequently the truth reference volumes were not visible to the eye (Figure 2, B3). The offsets, applied to the original intensity and contrast of the signal to attain the same values as the noise profile, were 47% (w.r.t. standard deviation) and 8.6% (w.r.t. mean). Next, the intensity and contrast of the signal were adjusted to above and below this mean value to determine the effective detection limits of each of the filters. In case II and case IV (Figure 2 A2-E2, A4-E4) the signal intensity was set to 0.43% above and below the mean of the noise. Case I and case V (Figure 2 A1-E1, A5- E5) were less stringent cases in which the signal intensity was 0.72% above and below the level of noise. It should be noted that the difference between C1-E1 and C5-E5 is that the particle contrast has been inverted (black to white, respectively).

The results of these analyses provided a clear indication of the limitations of each of the three filters tested. Unsurprisingly, for case III (perfectly overlayed noise and signal), none of the three filters were able to distinguish signal from noise (nor did any of the three filters show any detection artefacts). In all other cases, the 3D BLE filter performed at least as well or better than both the 3D recursive and Canny algorithms.

At the less stringent settings (C1-E1; C5-E5) all three filters detected 4 and 8 pixel wide edges. The Canny filter appeared to be more affected by background noise (Figure 2 C1 vs E1; C5 vs E5) but also at the cost of artifactual noise contamination of the edge. It did however detect 2 pixel edges better. In contrast the 3D BLE filter yielded clearly defined and relatively noise-free contours but resolved the 2 pixel edges less well. Connectivity could be increased manually in the 3D BLE filter by raising the threshold setting from 0.85 (see Eq. 5) but at a cost of increased background noise. Overall, this suggested that the performance of the Canny and 3D BLE filters was similar.

At the most stringent intensity test settings (B2-E2; B4-E4) the 3D BLE and Canny filters both performed significantly better than the 3D recursive filter. The Canny and 3D BLE filters both effectively detected 4–8 pixel wide edges. Again the Canny filter yielded a level of performance better than 3D BLE but at the cost of increased artifactual noise contamination of the edge. In terms of the detection of 2 pixel edges, the 3D BLE and Canny filters were both close to their limits of detection. Generally the 3D BLE appeared to be better at suppressing noise while the Canny appeared better at detecting 2 pixel edges, but the differences were minimal.

Performance of the three filters was quantified by calculating RMSE values (see Table 2) between the control/truth volume constructed by applying the Canny edge-detector to the noise/CTF/envelope-free variant of Figure 1-A1 and the filtered output volumes (Figure 2 C–E). The 3D Canny outperformed the 3D BLE when the signal mean was offset ±0.43% from the noise, but failed to recover the edge information in all cases, especially at an object width of 2 pixels and signal offset of −0.43%. The 3D BLE outperformed the 3D recursive at ±0.43%, but performed roughly equal to the 3D recursive at ±0.72% with minor differences in RMSE scores.

**Table 2:** Statistical evaluation of filter performance using synthetic volumes contaminated with different levels of simulated experimental noise shown in Figure 2.^a^

NoiseSignal mean above noise mean	3D BLE (%)	3D recursive (%)	3D Canny (%)
**−0.72**	48.8 (3.66)	44.47 (3.04)	48.61 (3.63)
**−0.43**	65.13 (6.52)	73.08 (8.21)	48.82 (3.66)
**0**	99.48 (15.22)	80.42 (9.95)	58.67 (5.29)
**0.43**	65.51 (6.60)	71.22 (7.80)	58.89 (5.33)
**0.72**	49.28 (3.74)	44.64 (3.07)	53.82 (4.46)

a RMSE scores between the input volumes and the three filter outputs are shown in bold. Smaller scores represent higher levels of correlation with the input volume. Values in brackets are the percentage voxel variation between input volumes and the three filtered outputs.

### Application to biological test data

**Figure 3:**
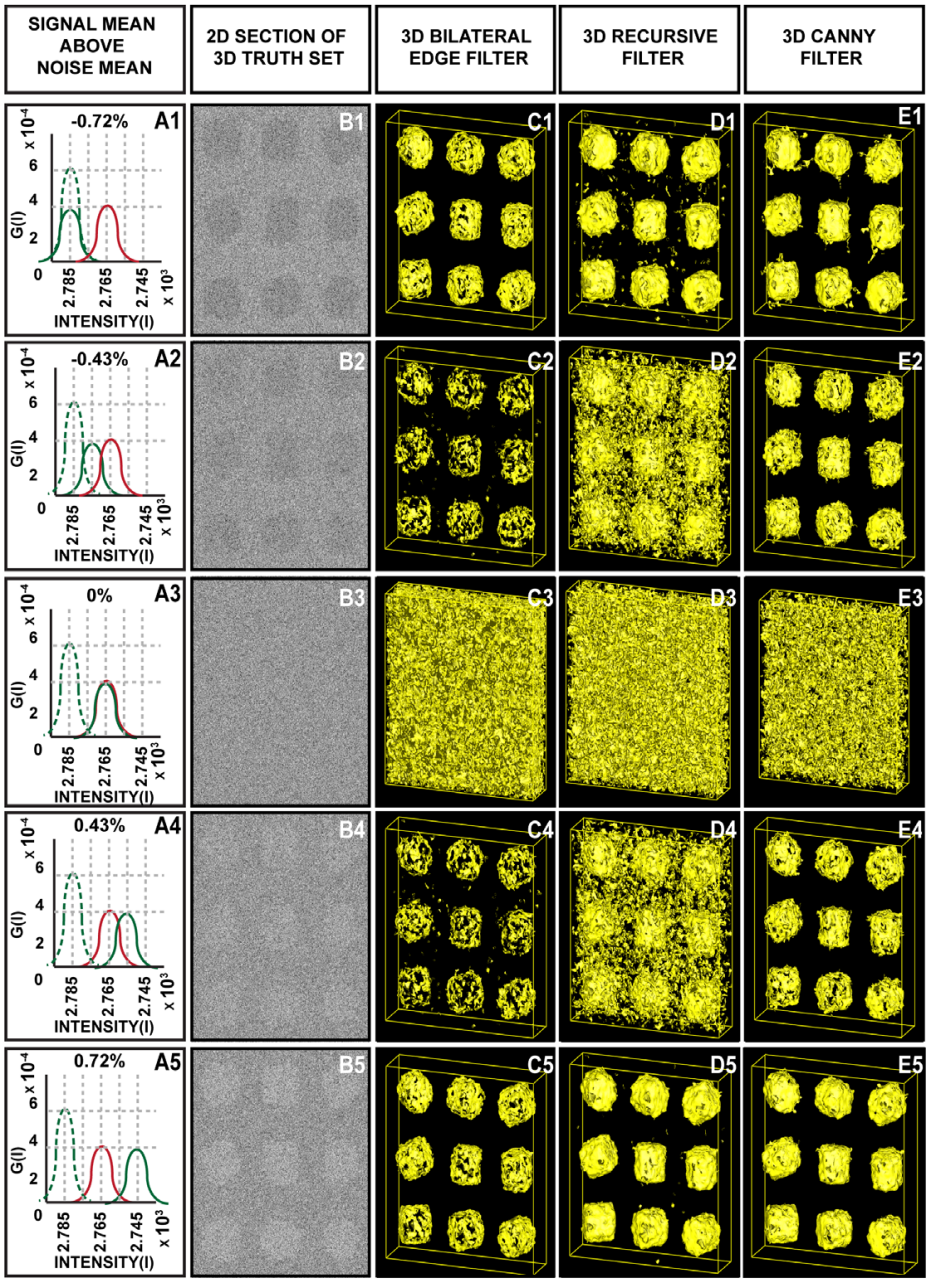
Detection of molecular volumes using 3D BLE. The ability of the3D BLE, 3D recursive and 3D Canny filters to resolve molecular contours was assessed using a test volume populated with 3D GroEL molecules. A representative region of the test volume showing 9 molecules is shown. (**A1–A5**) SNR illustrated as for Figure 2. (**B1–B5**) 2D sections taken from synthetic volumes contaminated with experimental noise. (**C1–C5**) Surface rendering of results following application of the 3D BLE filter applied to the test volume contaminated with experimental noise. (**D1–D5**) Surface rendering of 3D recursive-filtered test volume. (**E1–E5**) Surface rendering of 3D Canny-filtered test volume.

Having evaluated the performance of the 3D BLE filter using a synthetic truth reference set comprised of simple geometric shapes, we next used a simulated cryo-tomogram populated with 100 uniquely oriented copies of the GroEL chaperonin complex to evaluate the performance of the filter in a more biologically relevant context. Figure 3 demonstrates the performance of the three filters on a representative area of this simulated cryo-tomogram [22] containing nine GroEL molecules in different orientations. The first column (Figure 3 A1–A5) shows the relative ratio and offset of the mean signal contrast compared to the background noise (as in Figure 2 A1–A5). Upon close inspection, the 3D BLE (Figure 3 C1–5) and 3D Canny (Figure 3 E1–5) filters showed better edge connectivity than the 3D recursive (Figure 3 D1–5). In addition, the 3D recursive filter started to become less effective at removing noise at ±0.43% noise over signal (Figure 3 D2, D4). The 3D BLE filter, with fully automated parameter optimisation, performed nearly as well as the 3D Canny. Differences in the performance of the 3D BLE and Canny filters were attributed to connectivity of additional noise in the Canny images and lower detection of 2 pixel edges in the BLE. Overall therefore the BLE and Canny again seemed similar in performance, but the fully automated 3D BLE filter provided a considerable processing advantage.

### Application to experimental data

**Figure 4:**
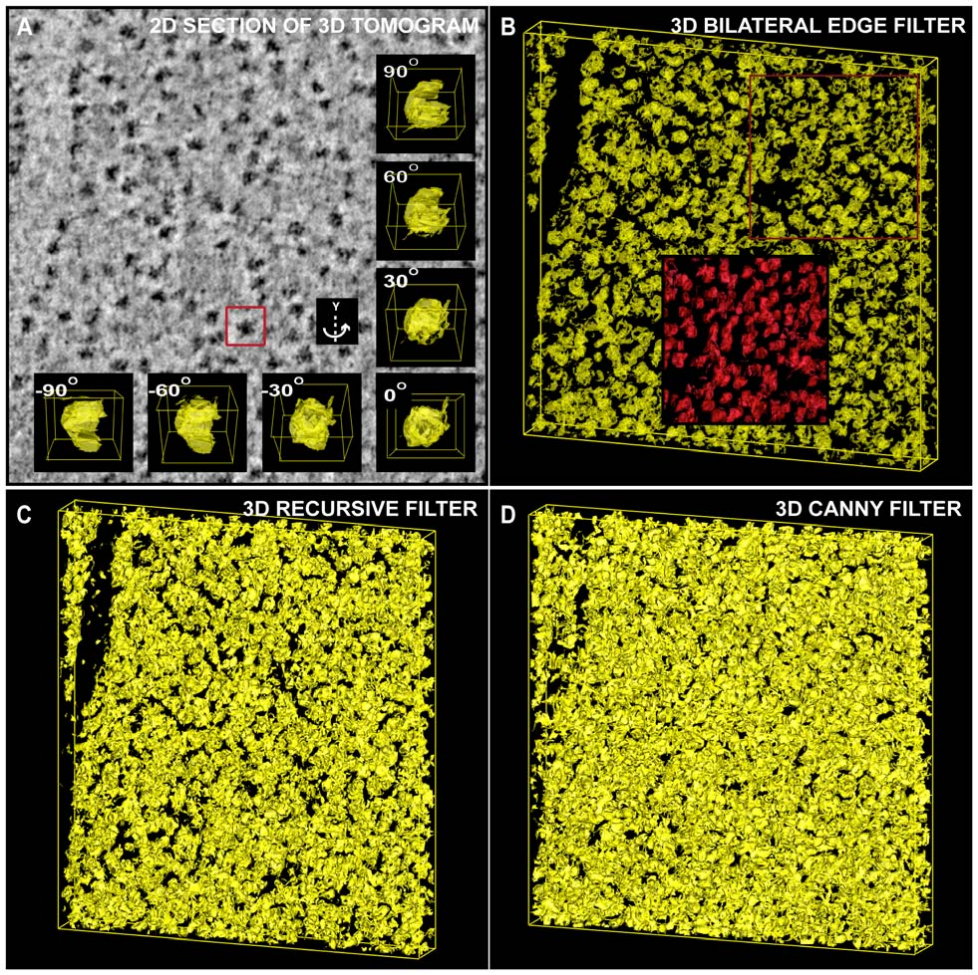
Extraction of molecular contours from an electron tomogram subvolume. Application of the 3D BLE, 3D recursive and 3D Canny filters to a subvolume of an experimentally-recorded tomogram of a resin-embedded *C. reinhardtii* cell. (**A**) Unprocessed, central 2D cross-section of the subvolume extracted from the 3D tomogram showing a region of the chloroplast heavily populated with putative macromolecular assemblies (dark objects). The inset in (A) highlights a randomly chosen single particle, represented as an isosurface rendering and shown at a selected number of orientations around the y-axis. (**B**) 3D surface rendering of results obtained from application of the 3D BLE filter. (**C**) Surface rendering of the 3D recursive-filtered subvolume. (**D**) Surface rendering of the 3D Canny-filtered subvolume.

We compared the ability of the 3D BLE, 3D recursive and 3D Canny filters to extract molecular edge contours from an 800×800×100 voxel region of a dual tilt tomographic reconstruction recorded from a resin-embedded, sectioned and post-stained *C. reinhardtii* cell (Figure 4). The dark densities are putative macromolecular assemblies, having an approximate diameter of 25 nm – roughly equivalent to the size of a ribosome. In this test, the 3D BLE filter (Figure 4B) clearly outperformed both the 3D recursive (Figure 4C) and 3D Canny edge detector (Figure 4D). Noise suppression in the 3D BLE-filtered image (Figure 4B) was considerably enhanced and contours around the putative macromolecular particles are thus more accurate and less corrupted by spurious noise densities. We concluded that the most likely reason for the 3D BLE filter outperforming the Canny and 3D recursive filters in this test was that the SNR of the input data (Figure 4A) was significantly higher than that of any of the other test images (see Figure 1, 3). This was consistent with the enhanced performance of the 3D BLE filter observed under high SNR conditions in all of the previous tests (see Figure 1, 2; Tables 1, 2). We surmised that the 3D BLE behaved better by detecting more true positive edges and suppressed more noise for this tomographic data set, and that it again achieved this using a fully automated algorithm.

The ultimate aim of running an edge-detection algorithm is to obtain high quality and continuous 3D contours. Noise can however result in discontinuities. To enhance the performance of the 3D BLE filter further in this regard, the edges that it detected were completed using a Bspline interpolation (Figure 4B-inset). The use of the Bspline clearly improved connectivity. This is seen at the molecular scale in Figure 4A (insets) where one of the segmented particle volumes is shown in a range of orientations. This experiment demonstrated that the accuracy of the molecular contours obtained was high and sufficient for the detection of individual macromolecular assemblies within experimentally-recorded electron tomograms. This potentially paves the way for automated detection and extraction of molecular volumes for downstream 3D alignment, classification and single particle averaging.

### Automation of the development of a molecular resolution cellular 3D atlas

In the preceding sections we established that the automated 3D BLE filter could be applied to recover molecular-level detail from noise-corrupted and real experimental volumes. In particular we demonstrated the application of the 3D BLE filter to the accurate and automated segmentation of macromolecular structures *in situ*. While the examples used in the preceding sections were based on real tomographic data, the detection of individual particles was not complicated by the presence of neighboring structures that might have confounded its performance. As a final test, we therefore applied the 3D BLE algorithm to a larger subvolume packed with potentially confounding macromolecular and organellar structures. For this test, we extracted a subvolume from a tomogram encompassing a large cytoplasmic volume imaged from a murine pancreatic cell that had been analysed in detail by manual segmentation in a previous study [23]. This set was chosen because it contained tightly packed molecular and organellar contours and because the performance of the 3D BLE could be compared to that achieved by manual segmentation performed as part of the original study.

**Figure 5:**
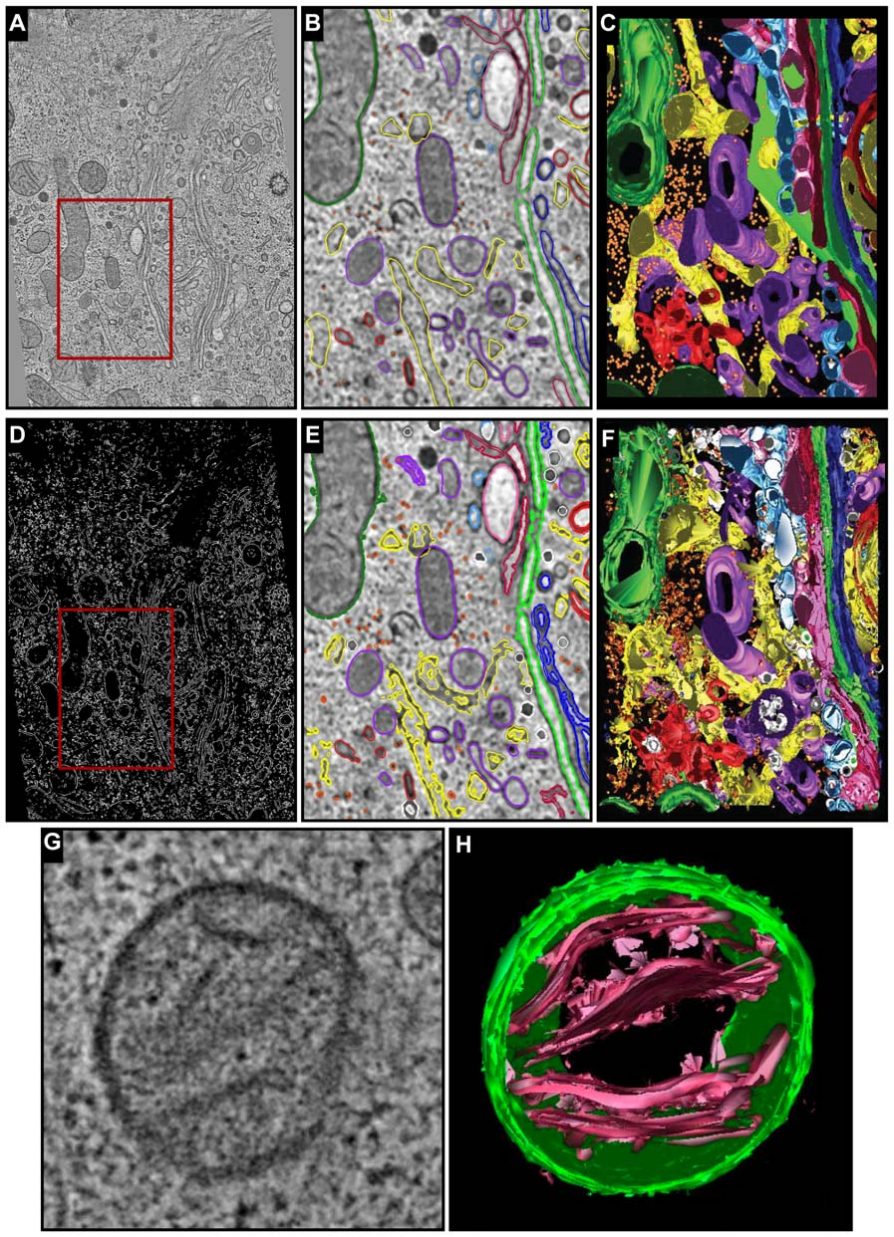
Segmentation of the Golgi region of an insulin-secreting pancreatic beta cell line HIT-T15. (**A**) A tomographic slice (slice 33) extracted from the reconstructed volume reported in [23]. The region demarcated by a red box is shown in (B). (**B**) Objects were segmented by manually drawing colored lines (contours) using IMOD. (**C**) Surface-rendered 3D model of the Golgi region analysed in (B) by manual segmentation. (**D**) 3D BLE-filtered tomogram. (**E**) Contours detected automatically by the 3D BLE were then manually colored for comparison to the manually segmented volume shown in (B). (**F**) Surface-rendered 3D model generated by automatic segmentation of the same region shown in B. Coloring in (C–D) and (E–F) is as follows: the seven cisternae that comprise the Golgi in the region - C1, light blue; C2, pink; C3, cherry red; C4, green; C5, dark blue; C6, gold; C7, bright red. ER, yellow; membrane-bound ribosomes, blue; free ribosomes, orange; mitochondria, bright green; dense core vesicles, bright blue; clathrin-negative vesicles, white; clathrin-positive compartments and vesicles, bright red; clathrin-negative compartments and vesicles, purple; mitochondria, dark green. (**G**) A tomographic slice revealing the outer and inner membrane architecture of a mitochondrion in the Golgi region. (**H**) Surface rendering shows that automated 3D segmentation facilitated by the application of 3D BLE detects the mitochondrial membranes.

The results obtained following application of the 3D BLE to the pancreatic cell tomogram are shown in Figure 5. Figure 5A shows a representative 2D section of the 3D tomogram. Figure 5B shows the manually segmented structures reported by Marsh *et al.* [23] for a representative subvolume (demarcated by the red box in Figure 5A) of the full 3D tomogram. Figure 5C shows a 3D surface view of Figure 5B. Figure 5D shows the output obtained following processing of the complete volume with the 3D BLE filter. Figure 5E shows the corresponding densities detected using the 3D BLE filter. A comparison of Figure 5B and E indicates that 3D BLE filter was able to detect all of the structural elements identified by manual segmentation in a fully automated manner. The tomographic slice in Figure 5G (which is extracted from the dataset used in Figure 5A) shows the membrane organization of a mitochondrion in the region. Figure 5H shows the surface view of the outer membrane as well as the inner cristae (pink). The detection of inner cristae clearly highlights the capability of the 3D BLE. By comparison, manual segmentation is labor-intensive and requires some biological expertise.

These results indicated that at the organellar level, the 3D BLE was capable of extracting structural data from cellular electron tomograms in an automated manner. The speed with which this was achieved was another important property of this filter. The 3.1×3.2×1.2 µm^3^ volume reported by Marsh *et al.* [23] required around 9–12 months to segment manually using IMOD [24]. The 3D BLE detected all of these structures (see Fig. 5F, full volume) in 1 h 53 min, which is approximately 4000× faster. It should also be noted that the 3D BLE additionally detected a much more extensive set of particle contours (Figure 5D), but only those that corresponded to the set of previously reported manual contours were shown, in order to facilitate a direct comparison. The current implementation of the 3D BLE required that the detected contours corresponding to those reported by Marsh *et al.* [23] be marked up (i.e. colored) manually, a process which remains time consuming. In the future however the use of algorithms such as those reported by Woolford *et al.* [13] could be used to detect and therefore classify particles/organelles based on size and shape to establish rules for a first pass of automated contour classification prior to manual curation.

### Evaluation of computational requirements

In addition to noise suppression and true edge accuracy, an important consideration in comparing the 3D BLE filter to current gold standard filters was the consumption of computational resources. Table 3 summarises the processing times and memory usage for the 3D BLE, 3D recursive and Canny filters. When considering the processing time required by the filter alone, the 3D BLE at first appeared to perform worst of all three filters, taking 63 s of CPU time to process the 385×512×128 voxel test volume shown in Figure 1, compared with 11 s for the 3D recursive filter and 21 s for the Canny. But the 3D BLE filter compared much more favourably, when taking into account the requisite pre- and post-processing steps. The 3D BLE filter reported here was implemented with a small, fixed filter window (5×5 local neighborhood - experimentally determined to minimize window size) with pre-processing and post-processing routines incorporated directly into the filter algorithm. In comparison, the 3D recursive filter required several pre-processing steps including the conversion of the input file to a raw image format as well as post-processing which included thresholding and conversion back to the original (in this example, MRC) image format. Taking these additional steps into account, the processing time of the 3D BLE (169 s) was less than that of the 3D recursive filter (186 s) and as has already been shown, yielded considerably improved results. The 3D BLE also required slightly less memory than the recursive filter when the resource requirements for file conversion and thresholding were taken into account (240.8 Mb vs ∼272 Mb).

**Table 3:** Comparison of processing resources consumed by each of the three filters evaluated using a synthetic volume (385×512×128 voxels) contaminated with different levels of Gaussian and impulse noise.

	CPU timeTotal^a^ (filter)sec	MemoryMb	No. of adjustableparameters
**3D BLE**	169 (63)	240.8	0
**3D recursive**	186 (11)	221.6 (272)	0
**3D Canny**	21^b^	485.0	3

a Total processing time includes pre-processing and post processing.

b Although the run time of the Canny filter is short, the process is not automated.

The Canny filter required no pre- or post-processing but unlike the 3D BLE and 3D recursive filters, which were fully automated, the Canny edge detector required adjustment of three parameters (x: the standard deviation of the Gaussian, y: the high hysteresis threshold and z: the low hysteresis threshold). To evaluate all combinations of just two different values for each parameter would require 8 iterations of the filter. In practice we found that in the best cases, a minimum of 10 parameter combinations had to be tested to yield a result comparable in quality to that of the 3D BLE. Correspondingly, the effective processing time of the 3D Canny was increased at least 10-fold from 21 s to ∼210 s. This represents an increase of approximately 15–20% over and above the processing times of the 3D BLE and 3D recursive edge detectors ((210/187)×100 = 15%) when pre- and post-processing were taken into account, while making the process much more labor-intensive. Memory requirements (485 Mb) of the 3D Canny were also substantially higher – approximately double that of the 3D BLE. This highlights the value of the fully automated 3D BLE filter, in particular for high volume and/or high throughput image processing, where the one “adjustable” parameter (*σ*_2_) was automatically optimised in our implementation.

### Conclusion

We have described here a *bona fide* 3D implementation of the BLE filter that is able to accurately recover 3D contours describing the structure of individual macromolecular assemblies within real tomographic reconstructions of subcellular volumes. In these tests, the ability of the 3D BLE to accurately localize and detect edges in conjunction with noise suppression has been demonstrated. The performance meets or surpasses that of computationally more expensive 3D edge detectors by providing a straightforward and automatable implementation that does not require manual parameter adjustment. It is especially well suited to 3D particle detection for subsequent volume extraction, 3D alignment and averaging and thus holds great promise for the rapid and accurate segmentation/identification of 3D macromolecular structures. The fact that the algorithm also yields contour information will likely prove advantageous for subsequent down-stream processing steps such as docking higher resolution structures determined from SPA, NMR, X-ray and electron crystallography *in situ* within lower resolution cellular tomograms.

## Methods

### Implementation

The 3D bilateral edge filter was developed in C++ using the BSoft C++ library [25], [26]. The code has been compiled and tested on Mac OS X operating systems (Snow Leopard). All testing and experiments were conducted on 3D volumes. Software and conceptual test data are available from the authors upon request.

### Test data/patterns

The accuracy and integrity of the implemented 3D BLE was initially tested using a truth set comprising a broad range of conceptual reference volumes representing different geometries (sphere, cylinder, triangular and rectangular prism) and edge widths (1, 2, 4, 8 or 16 pixels) designed to thoroughly test the response of the filter to curves, straight lines and directionality. The reference volumes were then sequentially corrupted with increasing combinations of impulse and Gaussian noise (5–80%), or with simulated experimental noise (see below). Filter performance was assessed in comparison to leading filters in the field including the 3D Canny [7] and 3D recursive filters [19].

Noise suppression and edge-detection capabilities of the 3D bilateral edge filter were also evaluated using an experimental test volume populated with one hundred uniquely oriented density maps generated from the 6 Å 3D reconstruction of GroEL (EMDB accession code 1081) [22]. In order to comprehensively evaluate filter detection limits, the test volume was contaminated with differing amounts of simulated experimental noise adjusted to achieve mean signal intensity either greater, equal or less than the mean noise intensity. Contrast variation was normally distributed by adjusting the signal mean intensity (−8.6% to +8.6%) to match that of the noise mean intensity. The width of the distribution was parameterised by the standard deviation of signal (−47% to +47%) to match that of the noise standard deviation, where 0% was the mean contrast of the embedded test objects.

The simulated experimental noise was extracted from cytosolic regions of an algal cell tomogram in which organelles, filaments or other major subcellular structures were absent. The intensity and contrast profiles of the noise were defined and this information was then used to model the noise profile in Figures 2 and 3. The signal strength of the truth reference particles was normalised relative to the noise i.e. the mean intensity and contrast of the truth reference images was set to the same value as the mean values of the noise so that particles were initially undetectable (See Figure 3 B3) and the contrast of the truth sets then adjusted in order to identify the maximum experimental noise tolerated by the three edge detectors.

Filter performance was evaluated in terms of true and false positive object detection rates and the calculated RMSE between the filtered volumes and the corresponding uncontaminated original volumes, as well as by comparison of processing times and memory requirements.

### Electron tomography

The final tests were performed on electron tomograms of either the chloroplast region of a *C. reinhardtii* cell or the Golgi region of an insulin-secreting pancreatic cell. For *C. reinhardtii*, cells (strain stm3 [27]) were prepared for plastic embedding by concentration, high pressure freezing then freeze substitution and fixation using 2% OsO_4_ (osmium tetroxide) and 1% TA (tannic acid) according to Jimenez *et al.* [28]. 300 nm sections were cut and post stained using 2% aqueous uranyl acetate and Reynolds lead citrate. Sections were imaged at 23,000× nominal magnification using a Tecnai F30 FEG-TEM (FEI) operating at 300 kV, equipped with a 4K×4K lens-coupled camera (Direct Electron). Tilt series data were collected over a range of ±60° at 1.5° increments along two orthogonal axes and recombined computationally to produce a dual-axis 3D reconstruction using the IMOD software package [23], [24], [29]. Experiments detailing the imaging, reconstruction and manual segmentation of the Golgi region of an insulin-secreting pancreatic cell are the focus of a separate study reported previously by Marsh *et al.* [23].
